# Blood pressure waveform contour analysis for assessing peripheral resistance changes in sepsis

**DOI:** 10.1186/s12938-018-0603-4

**Published:** 2018-11-20

**Authors:** Shaun Davidson, Chris Pretty, Joel Balmer, Thomas Desaive, J. Geoffrey Chase

**Affiliations:** 1 Department of Mechanical Engineering, University of Canterbury, Christchurch, New Zealand; 2 GIGA-Cardiovascular Sciences, University of Liège, Liège, Belgium

**Keywords:** Septic shock, Cardiovascular monitoring, Arterial pressure waveform, Pressure catheter, Linear least-squares

## Abstract

**Background:**

This paper proposes a methodology for helping bridge the gap between the complex waveform information frequently available in an intensive care unit and the simple, lumped values favoured for rapid clinical diagnosis and management. This methodology employs a simple waveform contour analysis approach to compare aortic, femoral and central venous pressure waveforms on a beat-by-beat basis and extract lumped metrics pertaining to the pressure drop and pressure-pulse amplitude attenuation as blood passes through the various sections of systemic circulation.

**Results:**

Validation encompasses a comparison between novel metrics and well-known, analogous clinical metrics such as mean arterial and venous pressures, across an animal model of induced sepsis. The novel metric *O*_*fe → vc*_, the direct pressure offset between the femoral artery and vena cava, and the clinical metric, ΔMP, the difference between mean arterial and venous pressure, performed well. However, *O*_*fe → vc*_ reduced the optimal average time to sepsis detection after endotoxin infusion from 46.2 min for ΔMP to 11.6 min, for a slight increase in false positive rate from 1.8 to 6.2%. Thus, the novel *O*_*fe → vc*_ provided the best combination of specificity and sensitivity, assuming an equal weighting to both, of the metrics assessed.

**Conclusions:**

Overall, the potential of these novel metrics in the detection of diagnostic shifts in physiological behaviour, here driven by sepsis, is demonstrated.

## Background

One of the leading worldwide causes of intensive care unit (ICU) admission and mortality is cardiovascular disease and dysfunction (CVD), which costs an estimated $863 billion USD per annum (2010), and is the cause of roughly 31% of global deaths (2013) [1]. Thus, research into mitigating these large human and economic costs is a significant and ongoing field, ranging from work on less invasively estimating useful, but difficult to measure, clinical metrics [2–5], or a lumped model of the cardiovascular system [6, 7], in an ICU environment, to work on investigating, selecting or estimating metrics of preclinical risk of cardiovascular [8, 9] and arterial disease [10, 11].

A notable contributor to the aforementioned human and economic costs is sepsis, a distributive shock condition that drives myocardial depression [12], is associated with 20–50% mortality in affected patients, and is, itself, responsible for some 10.4% of ICU admissions in the US [13]. Inadequate or incorrect diagnosis of conditions such as sepsis leads to increased length of stay, cost and mortality, further inflating the above figures [14, 15]. Management of CVD in the ICU is typically informed by measurements from catheters situated near the heart. Despite, or potentially partially due to, the information rich nature of their output waveforms, the use of these catheters is not necessarily associated with improved clinical outcomes [16, 17].

Specifically, the human mind can simultaneously track only three to four variables effectively [18]. The volume of information presented to clinicians in an ICU environment is often far greater than this number. Thus, simplification, abstraction, and synthesis to present the most relevant pieces of clinical information in an easy to interpret form, in real-time, has potential to provide significant benefits to patient diagnosis and titration of treatment.

The circulatory system can be broken down into pulmonary and systemic circulations. Due to the smaller diameter of arteries and veins in the pulmonary circulation, catheters are typically placed in the systemic circulation [19], popularly in the aorta, femoral artery or vena cava [20–22]. The catheter measured pressure waveform passing through the systemic circulation is modulated by the systemic circuit. Thus, the evolution of the blood pressure waveform contains information on the condition of the systemic circulation, and patient condition. There is potential to extract some of this information directly from these catheter pressure waveforms, without first having to model the entire circulatory system. This approach could offer significant insight for relatively minimal effort.

Several existing methods compare input and output waveforms without specific modelling of the associated systems. Transfer functions are among the most common, frequently generated using either fast Fourier transforms [23] or empirical mode decomposition [24]. While these two methods are adept at extracting information directly from waveforms, neither method is able to directly leverage the extensive a priori knowledge available on the circulatory system, making it more difficult to isolate specific physiological regions and influences in the waveforms, and thus making results more difficult to interpret as the result of specific physiological phenomena [25]. Further, both methods provide full spectra as an output for every set of heartbeats analysed, an output that is not necessarily any easier for a clinician to rapidly parse than the initial waveforms. Potentially due to these issues, most existing studies utilising transfer functions in cardiovascular medicine focus on longer term trends and the circulatory system’s auto-regulation mechanisms [26, 27], rather than intra-beat cardiovascular mechanics as in this study.

In contrast to the spectra output by transfer functions, clinical practice favours very simple, lumped approximations of the entire waveform, such as mean arterial pressure (MAP) and mean venous pressure (MVP) [19]. However, these simple metrics can fail to isolate different influences, such as breathing, or, in the case of venous pressure, downstream heart behaviour, that can make changes in these metrics more difficult to interpret as the result of specific physiological phenomena. Hence, the novel metrics produced must be readily and easily interpreted by clinical staff, while providing greater insight and ensuring physiological and clinical relevance.

This paper proposes a method for helping bridge this gap between the complexity of information available and the simple, lumped values favoured clinically. It focuses on comparing amplitude and mean values between analogous sections of the aortic, femoral and vena cava pressure waveforms. Similar to a transfer function, it is a direct comparison of an input to an output, but operates in the time domain and provides simple, lumped outputs instead of a full frequency spectrum. Method outputs are compared to well-known clinical metrics over an experimental data set of induced sepsis, a condition both clinically relevant, as previously mentioned, and diverse and difficult to diagnose [28, 29]. This method has the potential to provide outputs for easy, rapid interpretation in a clinical environment, while retaining more information from the underlying pressure waveforms than the well-known lumped parameters currently used in critical care.

## Methods

### Experimental data

All experimental procedures and protocols were reviewed and approved by the Institutional Animal Care and Use Ethics Committee of the University of Liège, Belgium (Reference Number 14-1726). Their guidelines conform completely with the Guide for the Care and Use of Laboratory Animals published by the US National Institutes of Health (NIH Publication No. 85-23, revised 1996), as well as EU DIRECTIVE 2010/63/EU on the protection of animals used for scientific purposes.

Five male, pure Piétrain pigs, weighing between 18.5 and 29 kg, were selected. Pigs are known to have similar cardiovascular physiology to humans, and in this weight range, Piétrain pigs typically produce similar stroke volumes and arterial pressures to adult humans, making them ideal for this type of experimental investigation [30]. The pigs were sedated, anesthetised and mechanically ventilated (GE Engstrom CareStation) through a tracheotomy, with a baseline positive end-expiratory pressure (PEEP) of 5 cmH_2_O. Proximal aortic (*P*_*ao*_), femoral (*P*_*fe*_) and central venous (*P*_*vc*_) pressures were continually sampled using pressure catheters (Transonic, NY, USA) with sampling rates of 250 Hz. Though the data was not employed in this study, a median sternotomy was used to access the heart, and an admittance P–V catheter (Transonic, NY, USA) placed in the left ventricle. The protocol included [3]:

- An induced model of septic shock using an endotoxin infusion (lipopolysaccharide from *E. coli*, 0.5 mg/kg) over 30 min, causing inflammatory responses, capillary leakage and decreased afterload, leading to hypovolemia, global tissue hypoxia, and cardiac failure [31]. As such, this model of septic shock would be expected to cause dramatic changes in the behaviour of the systemic circulation.
- Several PEEP driven recruitment manoeuvres (both pre- and post-endotoxin infusion), which drive a change in preload conditions and are typically associated with a decrease in mean blood pressure and cardiac output [32].
- One to four infusions of 500 mL saline solution over 30 min periods (both pre- and post-endotoxin infusion), simulating fluid resuscitation therapy, a key component of hemodynamic resuscitation in severe sepsis, which results in a change in circulatory volume [33–37].

Once sepsis was sufficiently progressed that the subject was near death, and the arterial and venous waveforms became drastically different and largely incoherent, information was discarded. Due to the extremely advanced stage of circulatory failure at which this occurred, this information is likely of no use for diagnostic ICU monitoring. Note that not all pigs completed the entire protocol due to varying reactions to the endotoxin infusion. As such, the length of the monitoring period varies. However, each dataset consists of at least 5000 heartbeats.

### Novel metric methodology

The method involves two distinct input–output comparisons. The first is between aortic pressure (*P*_*ao*_, input) and femoral pressure (*P*_*fe*_, output), encompassing the arterial system. The second is between femoral pressure (*P*_*fe*_, input) and vena cava pressure (*P*_*vc*_, output), encompassing both the arterial and venous systems, and the transfer between them. There are several reasons for the selections of these comparisons, both in a general ICU, and a sepsis context. First, all three locations are typical, well utilised sites for catheters in the ICU [20–22]. Second, comparing *P*_*ao*_ and *P*_*fe*_ allows isolation and evaluation of arterial dynamics, including arterial tone, which is known to change in sepsis [38]. Similarly, comparing *P*_*fe*_ and *P*_*vc*_ allows isolation and evaluation of the behaviour of the capillary beds, which are also known to be significantly influenced by sepsis [31]. Thus, these two comparisons isolate different components of the circulatory system that should be differently effected by sepsis, and will thus differently modulate the blood pressure waveforms passing through them as patient condition changes. Therefore, metrics derived from these comparisons, with other influences accounted for, should respond to and capture these sepsis driven dynamics.

#### From aortic pressure (*P*_*ao*_) to femoral pressure (*P*_*fe*_)

Both *P*_*ao*_ and *P*_*fe*_ are situated in the arterial half of systemic circulation, and have very similar waveforms [19]. The primary distinction between the waveforms is the dicrotic notch in *P*_*ao*_, which is a sharp dip in pressure due to the aortic valve shutting at the end of systole. Due to the elastic nature of the arterial system, this sharp dip has been largely smoothed out by the time the pressure wave reaches the femoral artery, and thus is typically not present in *P*_*fe*_. This difference must be accounted for when comparing the two waveforms. The process used to compare *P*_*ao*_ and *P*_*fe*_ is thus:

For every fourth heartbeat *i*:

1. Select *P*_*ao*_ and *P*_*fe*_ for a set of four heartbeats from beat *i* to beat *i *+ *3*. A set of four heartbeats corresponds to approximately one breathing cycle as set by a mechanical ventilator (15 bpm), and thus minimises fluctuations in the results due to applied pulmonary pressure [19].
2. Compare peak and trough timing of *P*_*ao*_ and *P*_*fe*_ to determine phase lag:
  1 $$\delta_{ao \to fe} = {\text{mean}}\left( {\left[ {\begin{array}{*{20}c} {\begin{array}{*{20}c} {t_{{{ \hbox{max} }(P_{fe} ,i)}} } & - & {t_{{{ \hbox{max} }(P_{ao} ,i)}} } \\ \vdots & & \vdots \\ {t_{{{ \hbox{max} }(P_{fe} ,i + 3)}} } & - & {t_{{{ \hbox{max} }(P_{ao} ,i + 3)}} } \\ \end{array} } \\ {\begin{array}{*{20}c} {t_{{{ \hbox{min} }(P_{fe} ,i)}} } & - & {t_{{{ \hbox{min} }(P_{ao} ,i)}} } \\ \vdots & & \vdots \\ {t_{{{ \hbox{min} }(P_{fe} ,i + 3)}} } & - & {t_{{{ \hbox{min} }(P_{ao} ,i + 3)}} } \\ \end{array} } \\ \end{array} } \right]} \right)$$
  where, for example, *t*_max*(Pfe,i)*_ represents the peak timing of the *ith* beat of *P*_*fe*_. *δ*_*ao → fe*_ is thus a positive value, in ms, representing the average amount of time by which *P*_*fe*_ lags *P*_*ao*_, based on the differences in min and max timing of these two waveforms.
3. Shift *P*_*ao*_ right (forward in time) by *δ*_*ao → fe*_ such that the peaks and troughs of *P*_*ao*_ are aligned with those of *P*_*fe*_. Henceforth, $\widehat{P}_{ao}$ refers to *P*_*ao*_ that has undergone this phase shift alignment.
4. Use linear least squares on analogous regions of the aligned $\widehat{P}_{ao}$ and *P*_*fe*_ to find relative amplitude and associated offset between waveforms:
  2 $$\left[ {\begin{array}{*{20}c} {\left( {\begin{array}{*{20}c} {\widehat{P}_{ao} \left( {t_{{{ \hbox{min} }\left( {\widehat{P}_{ao} ,i} \right)}} } \right)} \\ \vdots \\ {\widehat{P}_{ao} \left( {t_{{{ \hbox{max} }\left( {\widehat{P}_{ao} ,i} \right)}} } \right)} \\ \end{array} } \right)} & {\begin{array}{*{20}c} 1 \\ \vdots \\ 1 \\ \end{array} } \\ \vdots & \vdots \\ {\left( {\begin{array}{*{20}c} {\widehat{P}_{ao} \left( {t_{{{ \hbox{min} }\left( {\widehat{P}_{ao} ,i + 3} \right)}} } \right)} \\ \vdots \\ {\widehat{P}_{ao} \left( {t_{{{ \hbox{max} }\left( {\widehat{P}_{ao} ,i + 3} \right)}} } \right)} \\ \end{array} } \right)} & {\begin{array}{*{20}c} 1 \\ \vdots \\ 1 \\ \end{array} } \\ \end{array} } \right] \times \left[ {\begin{array}{*{20}c} {A_{ao \to fe} } \\ {\Delta_{ao \to fe} } \\ \end{array} } \right] = \left[ {\begin{array}{*{20}c} {\left( {\begin{array}{*{20}c} {P_{fe} \left( {t_{{{ \hbox{min} }\left( {\widehat{P}_{ao} ,i} \right)}} } \right)} \\ \vdots \\ {P_{fe} \left( {t_{{{ \hbox{max} }\left( {\widehat{P}_{ao} ,i} \right)}} } \right)} \\ \end{array} } \right)} \\ \vdots \\ {\left( {\begin{array}{*{20}c} {P_{fe} \left( {t_{{{ \hbox{min} }\left( {\widehat{P}_{ao} ,i + 3} \right)}} } \right)} \\ \vdots \\ {P_{fe} \left( {t_{{{ \hbox{max} }\left( {\widehat{P}_{ao} ,i + 3} \right)}} } \right)} \\ \end{array} } \right)} \\ \end{array} } \right]$$
  where relative amplitude is *A*_*ao → fe*_ and identification offset is *Δ*_*ao → fe*_. The waveform regions used in identification are highlighted in Fig. 1, and minimise the influence of the dicrotic notch on the linear least squares process.

**Fig. 1:**
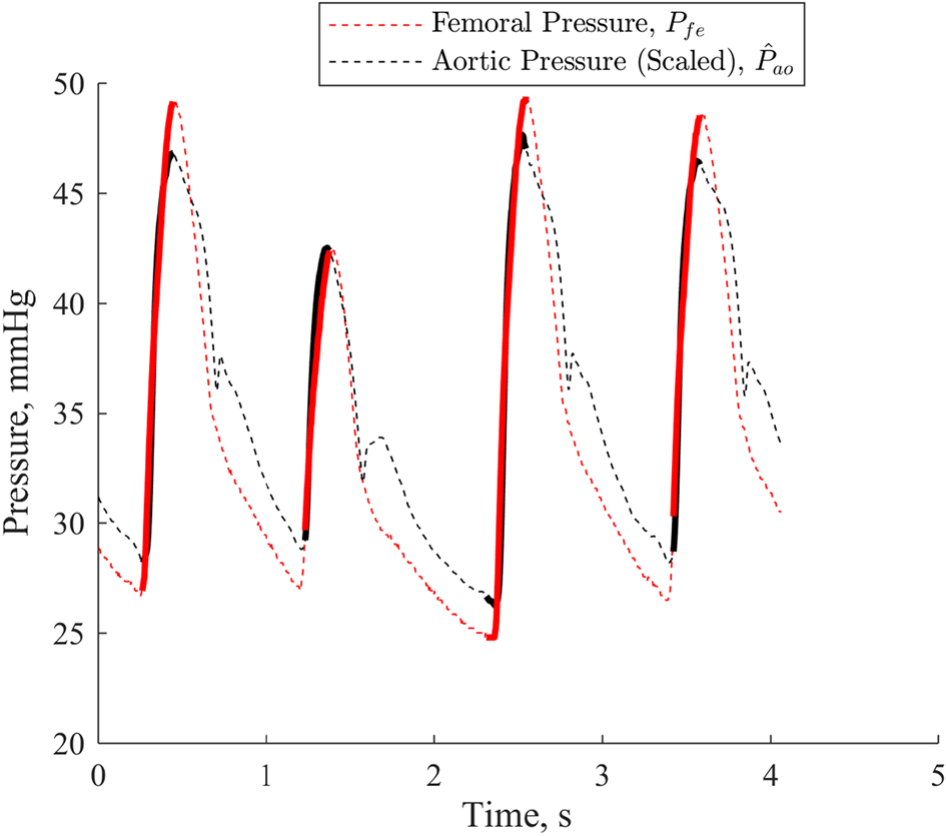
*P*_*ao*_ scaled, offset and phase shifted to match *P*_*fe*_. Solid, bolded regions of lines are those used in the linear least squares process
5. As *A*_*ao → fe*_ scales the entire *P*_*ao*_ waveform, thus modifying its mean value, *Δ*_*ao → fe*_ is heavily dependent on *A*_*ao → fe*_, and thus a poor indication of the actual pressure offset between the two waveforms. Thus, Eq. 3 is used to provide *O*_*ao → fe*_, a direct calculation of the mean difference between measured *P*_*ao*_ and the reconstructed *P*_*fe*_, and is defined:
  3 $$O_{ao \to fe} = {\text{mean}}\left( {\left[ {\begin{array}{*{20}c} {\widehat{P}_{ao} \left( {t_{{{\text{start}}\left( {\widehat{P}_{ao} ,i} \right)}} } \right)} & 1 \\ \vdots & \vdots \\ {\widehat{P}_{ao} \left( {t_{{{\text{end}}\left( {\widehat{P}_{ao} ,i + 3} \right)}} } \right)} & 1 \\ \end{array} } \right] \times \left[ {\begin{array}{*{20}c} {A_{ao \to fe} } \\ {\Delta_{ao \to fe} } \\ \end{array} } \right] - \left[ {\begin{array}{*{20}c} {\widehat{P}_{ao} \left( {t_{{{\text{start}}\left( {\widehat{P}_{ao} ,i} \right)}} } \right)} \\ \vdots \\ {\widehat{P}_{ao} \left( {t_{{{\text{end}}\left( {\widehat{P}_{ao} ,i + 3} \right)}} } \right)} \\ \end{array} } \right]} \right)$$

The resulting metrics characterising the relationship between the two pressure waveforms are the relative amplitude, *A*_*ao → fe*_, and the direct pressure offset, *O*_*ao → fe*_.

#### From femoral pressure (*P*_*fe*_) to central venous pressure (*P*_*vc*_)

Between the femoral artery and the vena cava sit the capillary beds of the systemic circulation, where vessel diameter, flow rate and pressure drop significantly [19]. The pulsatile blood pressure wave in *P*_*fe*_ ceases to exist in the capillaries, and *P*_*vc*_ is, instead, a result of upstream flow out of the capillaries and downstream right heart behaviour. In an input–output sense, *P*_*fe*_ drives flow in the capillaries which, in turn, is the upstream driver of *P*_*vc*_. While the direct pressure–pressure comparison from “Experimental data” section is a significant simplification of the relationship between *P*_*fe*_ and *P*_*vc*_, the capillary beds between the two waveforms are amongst the areas of the body most effected by sepsis, thus this easily achievable comparison is likely to be sensitive to sepsis. To interpret *P*_*vc*_ as the result of upstream behaviour, as much of the downstream right heart influence on the waveform must be filtered out as possible.

**Fig. 2:**
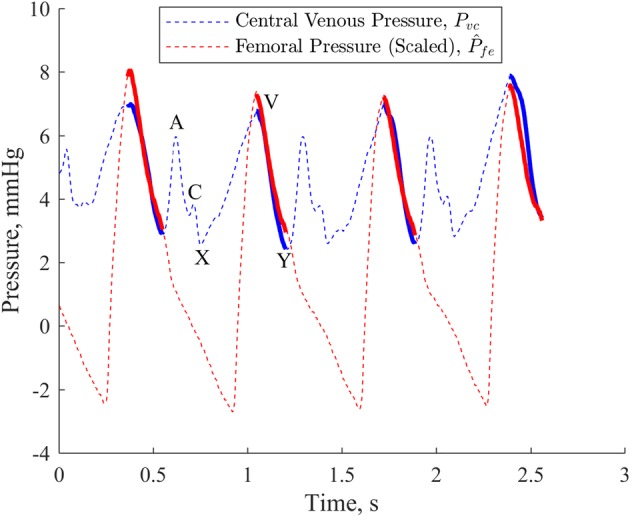
*P*_*fe*_ scaled, offset and phase shifted to match *P*_*vc*_. Solid, bolded regions of lines are those used in the linear least squares process. ‘A’, ‘C’ and ‘V’ peaks as well as ‘X’ and ‘Y’ troughs for a single beat are labelled

Thus, the region between the ‘V’ peak and the ‘Y’ trough of *P*_*vc*_ is used for linear least squares, as shown in Fig. 2, while the ‘A’ peak and ‘C’ peaks, which are the results of active downstream atrial and ventricular behaviour [19], are ignored. The ‘V’ peak can typically be identified as the peak sitting in the latter half of the *P*_*vc*_ waveform for a given heartbeat, and the ‘Y’ trough as the trough directly following the ‘V’ peak.

While pulsatile pressure is not directly transferred across the capillaries, mean blood pressure passes through in an attenuated form. To compare the upstream and downstream pressure behaviour beat-by-beat, lower frequency influences such as breathing should be removed or adjusted for where possible. Here, this is accomplished by aligning the underlying mean components of the two waveforms, helping to minimise the influence of this lower frequency behaviour on the pressure–pressure comparison. Thus, the *i*th beat of *P*_*fe*_, *P*_*fe,i*_, is set to correspond instead to *P*_*vc,i*+*j*_, where *j*, is some positive shift that solves the optimisation problem:

4 $$j = {\text{argmin}}_{j} \left\| {\frac{{\Delta { \hbox{max} }\left( {P_{vc,i + j} } \right)}}{{\Delta \left( {i + j} \right)}} - \frac{{\Delta { \hbox{max} }\left( {P_{fe,i} } \right)}}{\Delta i}} \right\|_{2}$$

Equation 4 yields the value of *j* providing the best agreement in directional peak to peak variation between *P*_*vc*_ and *P*_*fe*_. This process functions by aligning the inter-beat *P*_*fe*_ and *P*_*vc*_ waveforms based on their response to lower frequency influences. Even though *j* is not constrained in Eq. 4, it is consistently found to be equal to 3 or 4, which supports the validity of this approach, and yields a consistent and reasonable result.

Thus, the final process used to compare *P*_*fe*_ and *P*_*vc*_ is defined, for every fourth heartbeat, *i*:

1. Select four heartbeats for both *P*_*fe*_ and *P*_*vc*_, from the beginning of beat *i* to the end of beat *i *+ *3* for *P*_*fe*_ and from the beginning of beat *i *+ *j* to the end of beat *i *+ *j *+ *3* for *P*_*vc*_, where *j* is the subject specific number of heartbeats’ time it takes for a pressure wave to pass through the systemic circulation.
2. Compare peak and trough timing of *P*_*fe*_ and *P*_*vc*_ to determine phase lag. Note this lag is additional to the lag already implemented by the term *j*:
  5 $$\delta_{fe \to vc} = {\text{mean}}\left( {\left[ {\begin{array}{*{20}c} {\begin{array}{*{20}c} {t_{{{ \hbox{max} }(P_{vc} ,i + j)}} } & - & {t_{{{ \hbox{max} }(P_{fe} ,i)}} } \\ \vdots & & \vdots \\ {t_{{{ \hbox{max} }(P_{vc} ,i + j + 3)}} } & - & {t_{{{ \hbox{max} }(P_{fe} ,i + 3)}} } \\ \end{array} } \\ {\begin{array}{*{20}c} {t_{{{ \hbox{min} }(P_{vc} ,i + j)}} } & - & {t_{{{ \hbox{min} }(P_{fe} ,i)}} } \\ \vdots & & \vdots \\ {t_{{{ \hbox{min} }(P_{vc} ,i + j + 3)}} } & - & {t_{{{ \hbox{min} }(P_{fe} ,i + 3)}} } \\ \end{array} } \\ \end{array} } \right]} \right)$$
  *t*_max(*Pvc*)_ refers specifically to the ‘V’ peak of the waveform, and *t*_min(*Pvc*)_ to the ‘Y’ trough. *δ*_*fe → vc*_ is once again a positive value as defined, in ms, this time representing the amount of time by which *P*_*vc*_ lags *P*_*fe*_.
3. Shift *P*_*fe*_ right by *δ*_*fe → vc*_ such that the peaks and troughs of *P*_*fe*_ are aligned with those of *P*_*vc*_. Henceforth, $\widehat{P}_{fe}$ refers to *P*_*fe*_ that has undergone this phase shift, and is thus aligned with *P*_*vc*_.
4. Use linear least squares on analogous regions of $\widehat{P}_{fe}$ and *P*_*vc*_ to find:
  6 $$\left[ {\begin{array}{*{20}c} {\left( {\begin{array}{*{20}c} {\widehat{P}_{fe} \left( {t_{{max\left( {P_{vc} ,i + j} \right)}} } \right)} \\ \vdots \\ {\widehat{P}_{fe} \left( {t_{{min\left( {P_{vc} ,i + j} \right)}} } \right)} \\ \end{array} } \right)} & {\begin{array}{*{20}c} 1 \\ \vdots \\ 1 \\ \end{array} } \\ \vdots & \vdots \\ {\left( {\begin{array}{*{20}c} {\widehat{P}_{fe} \left( {t_{{max\left( {P_{vc} ,i + j + 3} \right)}} } \right)} \\ \vdots \\ {\widehat{P}_{fe} \left( {t_{{min\left( {P_{vc} ,i + j + 3} \right)}} } \right)} \\ \end{array} } \right)} & {\begin{array}{*{20}c} 1 \\ \vdots \\ 1 \\ \end{array} } \\ \end{array} } \right] \times \left[ {\begin{array}{*{20}c} {A_{fe \to vc} } \\ {\Delta_{fe \to vc} } \\ \end{array} } \right] = \left[ {\begin{array}{*{20}c} {\left( {\begin{array}{*{20}c} {P_{vc} \left( {t_{{max\left( {P_{vc} ,i + j} \right)}} } \right)} \\ \vdots \\ {P_{vc} \left( {t_{{min\left( {P_{vc} ,i + j} \right)}} } \right)} \\ \end{array} } \right)} \\ \vdots \\ {\left( {\begin{array}{*{20}c} {P_{vc} \left( {t_{{max\left( {P_{vc} ,i + j + 3} \right)}} } \right)} \\ \vdots \\ {P_{vc} \left( {t_{{min\left( {P_{vc} ,i + j + 3} \right)}} } \right)} \\ \end{array} } \right)} \\ \end{array} } \right]$$
  where relative amplitude is *A*_*fe → vc*_, and identification offset is *Δ*_*fe → vc*_. The waveform regions used in identification are highlighted in Fig. 2, and minimise the influence the upstream behaviour of the right atria and ventricle on the linear least squares process.
5. Once again, due to *A*_*fe → vc*_ scaling the entire *P*_*fe*_ waveform, and thus modifying its mean, *Δ*_*fe → vc*_ provides a poor indication of the actual pressure offset between *P*_*fe*_ and the reconstructed *P*_*vc*_. Thus, Eq. 7 is used to provide *O*_*fe → vc*_, a direct calculation of the mean difference between *P*_*fe*_ and the reconstructed *P*_*vc*_.
  7 $$O_{fe \to vc} = {\text{mean}}\left( {\left[ {\begin{array}{*{20}c} {\widehat{P}_{fe} \left( {t_{{{\text{start}}\left( {\widehat{P}_{fe} ,i} \right)}} } \right)} & 1 \\ \vdots & \vdots \\ {\widehat{P}_{fe} \left( {t_{{{\text{end}}\left( {\widehat{P}_{fe} ,i + 3} \right)}} } \right)} & 1 \\ \end{array} } \right] \times \left[ {\begin{array}{*{20}c} {A_{fe \to vc} } \\ {\Delta_{fe \to vc} } \\ \end{array} } \right] - \left[ {\begin{array}{*{20}c} {\widehat{P}_{fe} \left( {t_{{{\text{start}}\left( {\widehat{P}_{fe} ,i} \right)}} } \right)} \\ \vdots \\ {\widehat{P}_{fe} \left( {t_{{{\text{end}}\left( {\widehat{P}_{fe} ,i + 3} \right)}} } \right)} \\ \end{array} } \right]} \right)$$

The parameters of interest are the relative amplitude, *A*_*fe → vc*_, and the direct pressure offset, *O*_*fe → vc*_.

### Analyses

**Fig. 3:**
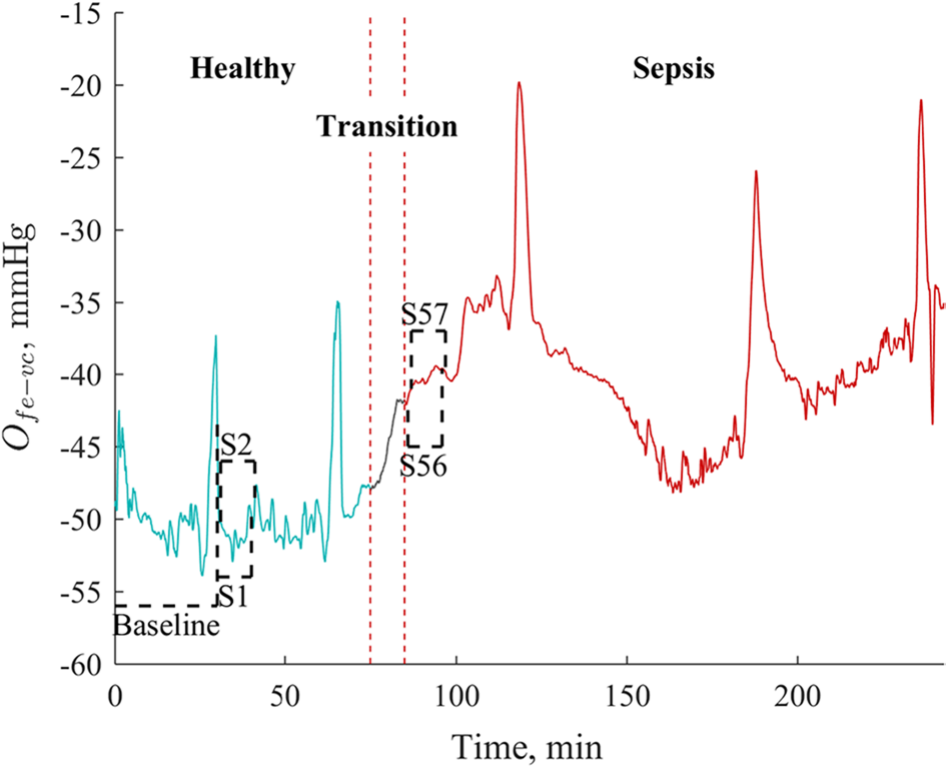
Sample regions used in developing ROC curves, including a 30 min baseline, healthy (S1, S2,…) and septic (S56, S57,…) rolling 10 min samples, with a 1 min shift in start point each time

The best comparison for the metrics developed in this paper, and which are currently employed in clinical practice, are MAP and MVP, as well as the difference between the two, ΔMP = MAP − MVP, a surrogate for systemic resistance [19] and broadly analogous to *O*_*fe → vc*_. Comparison between the novel and established metrics is largely based on response to induced sepsis expressed via Receiver Operator Characteristic (ROC) curves [39], which are designed to compare the diagnostic sensitivity and specificity or two or more metrics across various diagnostic thresholds. These ROC curves were generated through the following process, as shown in Fig. 3:

1. Take the median value for a given metric of a 30 min baseline sample (‘Baseline’ in Fig. 3) at the beginning of the recording
2. Take the median values for a given metric of rolling 10 min samples, excluding the baseline region, and with the beginning of each sample shifting by 1 min each time (‘S1’, ‘S2’, ‘S56’, ‘S57’ in Fig. 3).
3. Omit the samples involving the 10 min directly after endotoxin infusion, as this region is transitional cannot be easily characterised as ‘sepsis’ or ‘healthy’.
4. Categorise samples prior to endotoxin infusion as ‘healthy’ (‘S1’, ‘S2’ in Fig. 3), and thus a true negative/false positive, and post endotoxin infusion as ‘sepsis’ (‘S56’, ‘S57’ in Fig. 3), and thus a true positive/false negative.
5. Use a range of different baseline-sample median thresholds, *τ*, to develop the ROC curve.

Comparison between the ROC curve results determined for each novel and clinical metric encompassed three major elements:

1. A direct comparison of the ROC curves themselves, and the closeness of each curve to ‘perfect’ identifiability with a sensitivity and specificity of 1.0. This comparison illustrates the potential ability of each metric to differentiate between sepsis and healthy behaviour, across a variety of thresholds.
2. A comparison of the closeness to ‘perfect’ identifiability across all five pigs when the same relative threshold per metric was employed. Traditionally, aggregate ROC performance of a metric is evaluated by summing the area under the curve of each component plot, providing an indication of potential metric performance across thresholds and subjects. However, in this study, the desire is to assess each metric’s capability as limited by the clinical necessity to select a single a priori threshold, relative to a baseline median reading, which is applicable and effective across all subjects. Thus, this approach compares the averaged closeness to perfect identifiability achieved by each metric across all subjects for a given threshold, relative to subject specific baseline median values of said metric. The distance from ‘perfect’ identifiability employed in this approach was calculated using:
  8 $$\varepsilon \left( \tau \right) = \left\| {\left[ {\begin{array}{*{20}c} {\left( {1 - sensitivity\left( \tau \right)} \right)} \\ {\left( {1 - specificity\left( \tau \right)} \right)} \\ \end{array} } \right]} \right\|_{2}$$
  where *τ* represents the set of possible threshold choices, relative to a subject-specific baseline reading, for a given metric. Thus, *ε* is equivalent to the geometric distance from a given point on an ROC curve, for a given metric, to the top left-hand corner of the ROC axes, where sensitivity and specificity are given equal weighting. An example plot of *ε* for 4 demonstrative metrics is provided in Fig. 4.

**Fig. 4:**
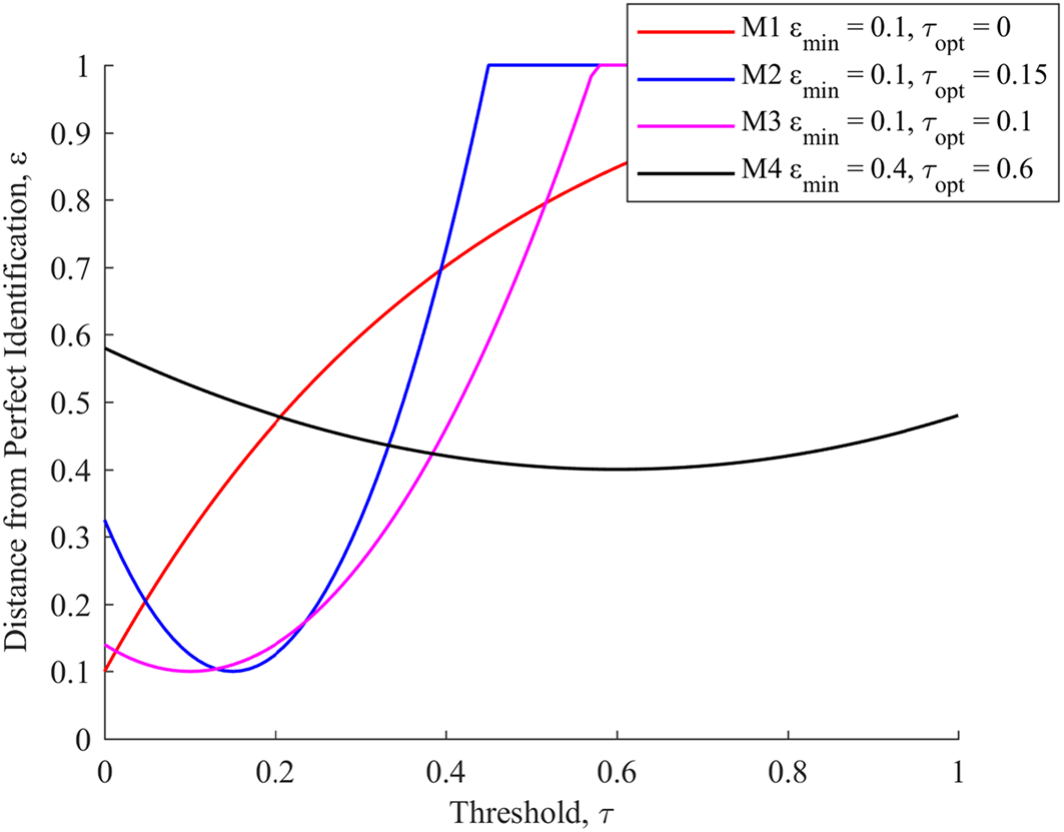
A set of ε curves for four demonstrative metrics. *ε*_min_ denotes the minimum distance from perfect identifiability, and *τ*_opt_ the threshold at which this occurs
  Of the four demonstrative metrics, metrics 1, 2, and 3 all have identical optimal *ε* values, and thus identical combined sensitivity and specificity performance at their respective optimum thresholds. However, metric 1, with an extremely low optimal threshold, is likely to generate a larger amount of false positives in a broader data set than the limited data set used for validation. Metrics 2 and 3 are more robust, with higher thresholds likely to prevent false positives due to random fluctuations in a broader data set. Of these two, metric 3 is superior to metric 2 due to the broader range of relatively low *ε* values, implying a lessened reliance of metric performance on picking the exact optimal threshold, as compared to metric 2. Finally, metric 4 demonstrates poor performance across the full range of thresholds trialled.
3. A comparison of how quickly after endotoxin infusion each metric reaches that metrics’ optimum threshold, corresponding to minimum *ε*, as established in the previous comparison. This comparison serves to illustrate how rapidly each metric can provide warnings about oncoming septic shock, which is of significant clinical importance [29].

Overall, this set of analyses provides a reasonably comprehensive picture of the clinical response of each metric to sepsis, as well as the sensitivity, specificity, and generalisability of this clinical response for use as a diagnostic tool.

## Results

**Fig. 5:**
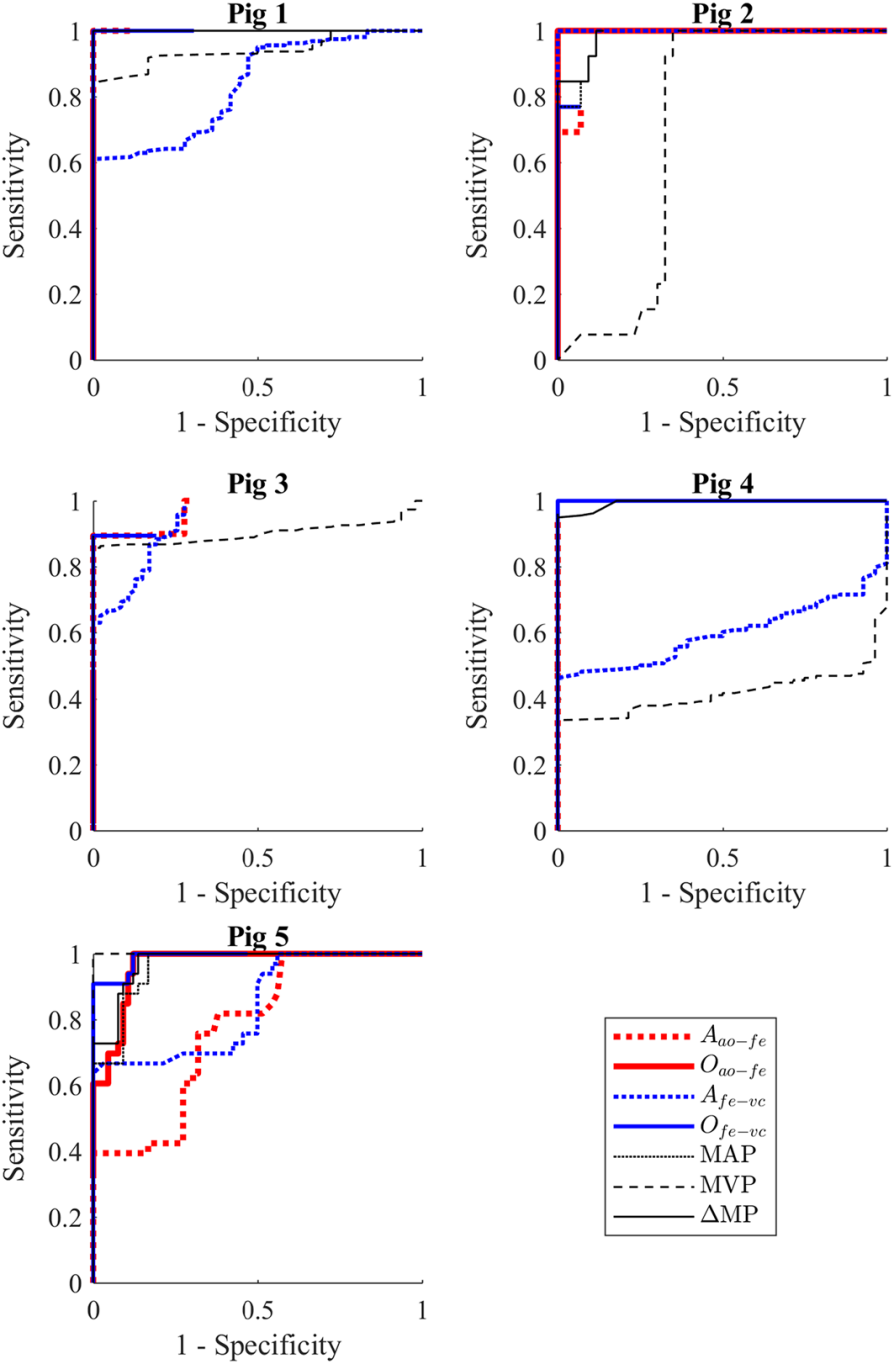
ROC plots for each pig, across all clinical and novel metrics

Figure 5 presents the ROC plots for the five pigs and seven metrics, which form the foundation of the comparisons between metrics presented in this paper. It is of note that perfect identifiability is achieved by various metrics for various pigs, and that there is notable variation in the performance of each metric across the pigs. Each ROC curve does not necessarily reach at the top right corner due to the use of each metrics’ baseline median reading, rather than a value of zero, as a comparator. When using the baseline median as a comparator, if pre-endotoxin metric values are both above and below the baseline median, no possible threshold will provide a specificity of 0, and if post-endotoxin metric values are both above and below the baseline median, no possible threshold will provide a sensitivity of 1.0.

Overall, *O*_*fe → vc*_ and the analogous ΔMP demonstrate consistently strong results, as do *P*_*ao*_ and *A*_*ao → fe*_. Conversely, *A*_*fe → vc*_, *O*_*ao → fe*_ and MVP, while displaying promising results for certain pigs, fail to consistently demonstrate strong results and thus are poor metrics for broad identification of sepsis. While the ROC plots serve to demonstrate the potential of a number of these metrics to serve as identifiers of sepsis, they do not provide clear, direct differentiation between the four metrics that appear to have the potential to perform well.

**Fig. 6:**
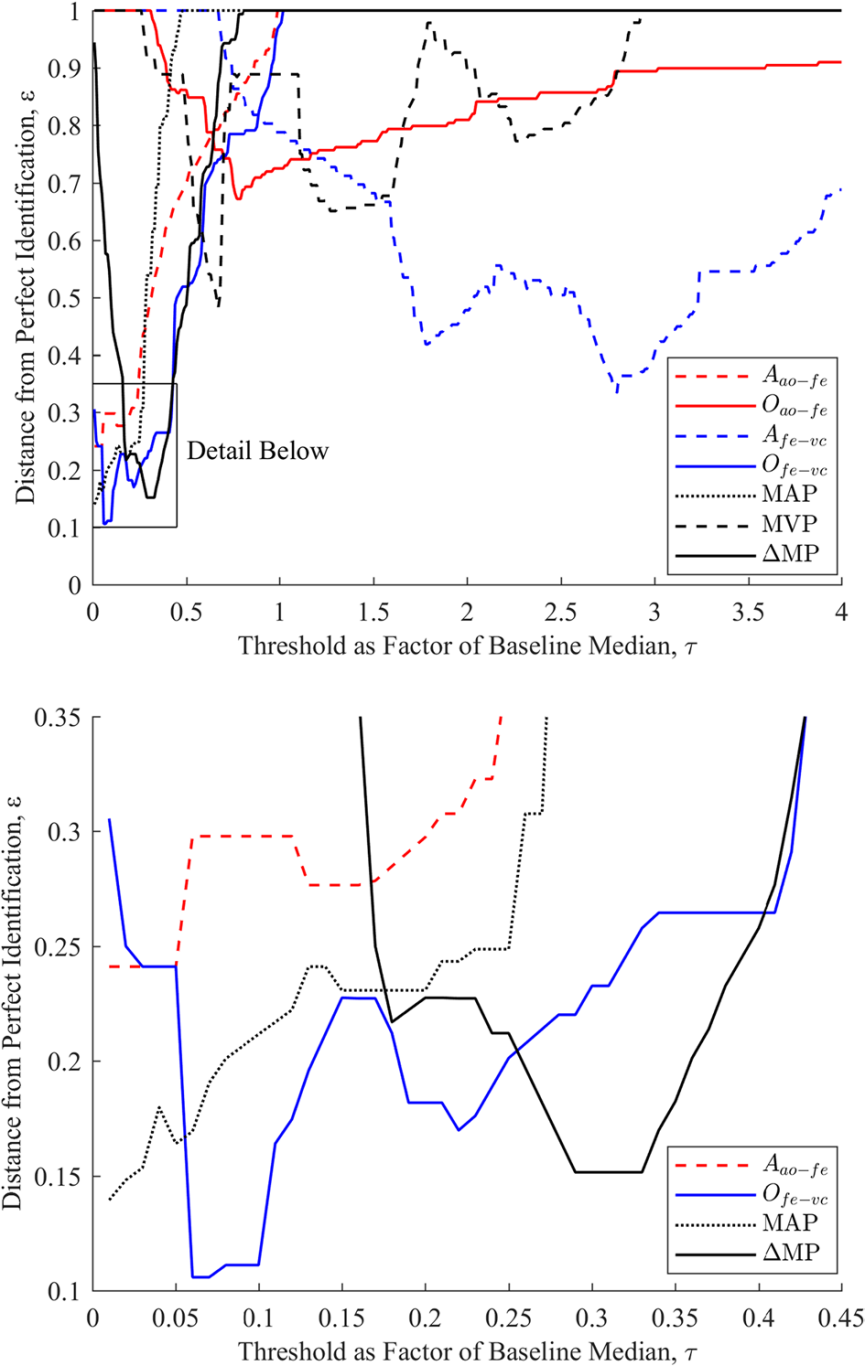
Distance from perfect identification (lower is better) achievable by each metric for a given threshold as a factor of baseline median

Figure 6 shows the closeness to perfect identifiability achieved by each metric for a given threshold relative to the subject specific baseline median reading across all pigs. Here, a clearer distinction between metric performances can be seen. Of the four metrics that performed well in Fig. 5, *O*_*fe → vc*_ delivers the overall best performance, at a threshold of 6–11% baseline median, followed by *MAP* at a threshold of 1%, ΔMP at a threshold of 29–33% and *A*_*ao → fe*_ at a threshold of 1–5% baseline median. *O*_*ao → fe*_, MVP, and *A*_*fe → vc*_ display poor performance, with no consistent, useful thresholds despite good performance on some individual pigs in Fig. 5.

Table 1 shows the time in minutes after endotoxin infusion at which each metric reaches the minimum optimal threshold presented in Fig. 6 and sepsis is declared, as well as the percentage of false positive results given during the monitoring period prior to endotoxin infusion. Here, an intuitive relationship between the rapid identification of sepsis, but a greater incidence of false positives for metrics with lower thresholds compared to metrics with higher thresholds is demonstrated, with ΔMP, *O*_*fe → vc*_, *A*_*ao → fe*_ and MAP sitting across the spectrum from conservative with a low rate of false positives, to non-conservative with a higher rate of false positives. Importantly, Table 1 omits false negatives occurring after the initial declaration of sepsis, which are included in Fig. 5, though these are of arguably less clinical value.

**Table 1:** The rapidity with which each metric reaches the minimum optimal threshold from Fig. 6, *t*, in minutes, and the percentage of false positives reported prior to endotoxin infusion, %FP

Pig	*A*_*ao → fe*_		*O*_*ao → fe*_		*A*_*fe → vc*_		*O*_*fe → nvc*_		*MAP*		*MVP*		*ΔMP*	
	*t*	%FP	t	%FP	t	%FP	t	%FP	t	%FP	t	%FP	t	%FP
Pig 1	9	0	168	0	168	0	8	0	1	36.1	8	19.4	33	0
Pig 2	1	7	1	0	14	0	17	0	1	11.6	22	0	22	0
Pig 3	3	29.8	52	0	131	0	31	0	38	0	134	0	68	0
Pig 4	24	0	1	100	148	0	1	3.6	17	0	1	100	94	0
Pig 5	1	36.4	1	15.2	29	0	1	27.3	1	100	8	0	14	9.1
Mean	7.6	14.6	44.6	23	98	0	11.6	6.2	11.6	29.5	34.6	23.9	46.2	1.8

## Discussion

### Clinical potential of novel metrics

Overall, the metrics novel metrics *O*_*fe → vc*_ and *A*_*ao → fe*_, as well as the clinical metrics MAP and ΔMP provide strong results in Figs. 5 and 6, demonstrating an ability to reach a relatively high sensitivity and specificity at a fixed, inter-subject sepsis detection threshold. The remaining three metrics appear to perform relatively poorly. Interestingly, MVP, despite being an output from the systemic circulation and capillary beds, which are primarily effected by sepsis, is a poor diagnostic. However, MVP appears to contribute positively to the metric ΔMP, which has similar performance and a considerably more conservative, but robust, threshold than the associated MAP.

As mentioned in “Analyses” section, the breadth of optimal regions in Fig. 6 is also of importance, as it indicates metric insensitivity to selection of a specific, appropriate threshold. *O*_*fe → vc*_, ΔMP, and *A*_*ao → fe*_ have reasonably broad optimal zones (6–11%, 29–33%, and 1–5%, respectively), suggesting some resilience to sub-optimal threshold selection. Note that the plot in Fig. 6 does not differentiate between whether the deviation from perfect identifiability of a metric is due to a loss of sensitivity or specificity, providing instead a combined metric with a 1:1 weighting between the two values. A separation of metric sensitivity and specificity is instead provided in Table 1.

From Table 1, arterial metrics MAP and *A*_*ao → fe*_, with their low 1% of baseline median thresholds, are highly sensitive, with mean times to sepsis detection of 11.6 and 7.6 min, respectively. However, this sensitivity comes at some cost in terms of specificity, with MAP and *A*_*ao → fe*_ having a false positive rate of 29.5% and 14.6%, respectively, prior to endotoxin infusion. Note that *A*_*ao → fe*_ appears clearly superior to MAP in Table 1, where false negatives after the initial declaration of sepsis are omitted, but not in Fig. 6, where these false negatives are included. The sensitivity of these arterial metrics to sepsis likely corresponds to a sepsis driven loss in arterial tone [38], resulting in significant pulse energy being lost in the arterial system.

Arterial-venous metrics are more conservative, trading some sensitivity for increased specificity. *O*_*fe → vc*_, with its threshold of 6%, has an average time to detection of 11.6 min and a mean false positive rate of 6.2%, and ΔMP with its threshold of 29% an average time to detection of 46.2 min and mean false positive rate of 1.8%. While the various metrics exist on a continuum without a clear ‘best’ metric across all categories, the results in Table 1 and Fig. 6 do make a very convincing case for the effectiveness of *O*_*fe → vc*_ as the superior metric, closely followed by ΔMP.

Sepsis is a fast acting condition [19], thus rapid diagnosis and treatment are highly important, and an increase in detection time from 11.6 min (*O*_*fe → vc*_) to 46.2 min (ΔMP) is significant relative to the time scale on which sepsis acts [29]. The relatively low, but distinct, threshold of 6–11% compared to the ΔMP threshold of 29–33% could suggest that the pressure–pressure comparison and filtering method used to derive *O*_*fe → vc*_ succeeds in removing the effects of right heart behaviour of MVP, providing a clearer picture of the underlying blood flow that has passed through the systemic circulation, and thus a clearer diagnostic picture. Reduction of the ΔMP threshold away from the optimal point to a similar false positive rate to *O*_*fe → vc*_ yields a 16.0 min detection time and 6.4% false positive rate at a 13% threshold, but also increases the false negative rate from 3.0 to 43.9%, as compared to the 9.0% false negative rate of *O*_*fe → vc*_. Thus, at this operation point, *O*_*fe → vc*_ provides a shorter detection time, as well as lower false positive and false negative rates, at negligible additional cost to ΔMP. The overall sensitivity and relative robustness of these arterial-venous metrics in detecting sepsis matches expectations, as the area between the aorta or femoral artery and vena cava includes the capillary beds and micro-circulation most affected by septic shock [29, 31], and supports the application of the pressure–pressure comparison approach to these waveforms.

It is worth noting that what loss in specificity is observed in the arterial-venous metrics is often driven by the mechanical ventilation RMs in the experimental protocol. Similarly, the relatively higher rate of false positives in *O*_*fe → vc*_ as compared to ΔMP are due to the fact that both the input (*P*_*ao*_) and output (*P*_*vc*_) for ΔMP lie in the thoracic cavity, and are thus both subject to an increase during a RM, thus partially cancelling each other out. In contrast, only the output (*P*_*vc*_) of *O*_*fe → vc*_ lies within the thoracic chamber, thus the RM increases output without directly influencing input, resulting in a more significant decrease in *O*_*fe → vc*_. It is important to note that, first, this decrease is physiological. Thus, the metrics are correctly representing underlying behaviour. Additionally, both metrics are largely able to avoid false positives due to this behaviour. Clinically, this specific issue might be managed by simply not reporting during RMs, in which case %FP for *O*_*fe → vc,*_ ΔMP, MAP and *A*_*ao → fe*_ fall to 1.0%, 0.0%, 21.8% and 7.2% respectively. However, the presence of false positives due to RMs illustrate in a broader sense a limited ability to distinguish between a decrease in pressure offset due to a relative increase in upstream pressure and a relative decrease in systemic resistance.

### Limitations

Overall, there are several limitations to this study that bear mention. First, and most importantly, one of the key difficulties in the diagnosis of sepsis is distinguishing sepsis from other disease states [28]. While the experimental data here is detailed, and includes a variety of clinically standard interventions alongside induced sepsis, it does not include non-sepsis disease states to provide a broader range of potential ‘false positive’ situations. This omission is an important consideration. Thus, the specificity results presented here more represent the ability of the method to distinguish between baseline and septic behaviour as opposed to sepsis and alternative disease states.

However, sepsis is typically diagnosed in the manner of a syndrome rather than a disease, with a reliance on the manifestation of a family of infection-driven symptoms, which typically respond to a family of treatments [29, 31]. This paper validates the ability of the presented methodology to accurately and rapidly identify one of these important signifiers of sepsis, associated with a sepsis driven fall in arterial tone as well as systemic resistance. Thus, while the methodology presented may not be posed to identify sepsis in isolation, it is capable of accurately and rapidly identifying metrics that can meaningfully contribute to the diagnosis of sepsis, along with other diseases [19, 29].

Further limitations include the study being limited to pigs, rather than humans, though this issue, as always, provides an excellent opportunity to explore an induced, controlled disease state with heavy instrumentation and provide a rigorous foundation for further studies translated to humans. The use of a baseline state is also a potential limitation, as patients on admission to an ICU are often not in a healthy, baseline circulatory state. However, once again, sepsis is representative of a series of symptoms and a further degradation of an unhealthy, but non-septic, circulatory system should be similarly diagnostic of sepsis as a degradation of a healthy circulatory system. Further, the baseline readings of the pigs varied considerably as the pigs had been anaesthetised, instrumented, and subject to a sternotomy, with varying response to these procedures, further accentuated by their highly varied response to the subsequent endotoxin infusion. Thus, there is some variability and thus some test of robustness in the methods reliance on a ‘healthy’ state baseline reading.

## Conclusions

The arterial metrics *A*_*ao → fe*_ (the relative pressure pulse amplitude between the aorta and femoral artery) and MAP were found to be highly sensitive to sepsis, providing rapid detection of the onset of symptoms, but at some cost in specificity. The arterial-venous metrics ΔMP and *O*_*fe → vc*_ (the direct pressure offset between the femoral artery and vena cava) were found to provide more robust, at a slight cost to sensitivity, detection of the onset of sepsis, with mean times to detection from endotoxin infusion of 46.2 min and 11.6 min respectively. Overall, the novel *O*_*fe → vc*_ provided the best combination of specificity and sensitivity, assuming an equal weighting to both, of the metrics assessed, followed by ΔMP. The novel metrics described in this paper do not require significantly more instrumentation to calculate than existing well-known and reported clinical metrics, and only minimal computational effort. Thus, these metrics have the potential to provide further, clinically relevant and easily interpretable information to clinicians at the patient bedside, for little to no additional clinical effort. Thus, the results in this paper provide a case for further evaluation of the clinical usefulness of these metrics, and their potential diagnostic benefits.
